# Temporally-Patterned Magnetic Fields Induce Complete Fragmentation in Planaria

**DOI:** 10.1371/journal.pone.0061714

**Published:** 2013-04-19

**Authors:** Nirosha J. Murugan, Lukasz M. Karbowski, Robert M. Lafrenie, Michael A. Persinger

**Affiliations:** 1 Department of Biology, Laurentian University, Sudbury, Ontario, Canada; 2 Behavioural Neuroscience Program, Laurentian University, Sudbury, Ontario, Canada; 3 Biomolecular Sciences Program, Laurentian University, Sudbury, Ontario, Canada; 4 Regional Cancer Program, Health Sciences North, Sudbury, Ontario, Canada; University of Zurich, Switzerland

## Abstract

A tandem sequence composed of weak temporally-patterned magnetic fields was discovered that produced 100% dissolution of planarian in their home environment. After five consecutive days of 6.5 hr exposure to a frequency-modulated magnetic field (0.1 to 2 µT), immediately followed by an additional 6.5 hr exposure on the fifth day, to another complex field (0.5 to 5 µT) with exponentially increasing spectral power 100% of planarian dissolved within 24 hr. Reversal of the sequence of the fields or presentation of only one pattern for the same duration did not produce this effect. Direct video evidence showed expansion (by visual estimation ∼twice normal volume) of the planarian following the first field pattern followed by size reduction (estimated ∼1/2 of normal volume) and death upon activation of the second pattern. The contortions displayed by the planarian during the last field exposure suggest effects on contractile proteins and alterations in the cell membrane’s permeability to water.

## Introduction

Whereas chemical effects upon life systems are determined by the complexity of spatial (molecular) structure, alterations by applied electromagnetic fields appear to be determined by the complexity and shape of the specific temporal pattern [1]. The traditional argument that powerful biological effects from weak magnetic fields would be minimal because of obscuration by intrinsic thermal variations (the “kT boundary problem”) may not be applicable to systems in non-equilibrium such as life forms [2]. While exploring the effects of exposure of planarian to a digitized complex, amplitude-modulated field that slows the growth rate of melanoma cells [3] *in vitro*, we combined two other complex-patterned, electromagnetic fields within the µT (microTesla) range that resulted in planarian being dissolved within a few hours of the exposure to the second pattern. We had never observed any phenomenon of such magnitude with these fields.

Planarians are optimal animals to assess the effects of weak, physiologically patterned magnetic fields in aqueous environments. Their neurons more closely resemble the neurons of vertebrates than even higher invertebrates [4]. Planarian are known for their large proportion of neoblasts, a stem-cell population with the potential to generate every cell type in the adult animal^5^. Their sensitivity to weak (earth magnitude) static magnetic fields has been known for decades [6]–[8]. Planarian capacity to regenerate and multiply asexually [9] is influenced by weak (∼10 µT) power frequency magnetic fields [10] and frequencies tuned to calcium resonance [11] which is a likely mechanism for membrane voltage-mediated changes in anterior gene expression [12]. Intensities as low as 40 nT and a variety of frequencies such as 1 Hz, 3 Hz, 7 Hz, 32 Hz and 60 Hz can stimulate fission [13].

Goodman et al [14] found that transected planarian exposed twice a day for one hour to weak 60 Hz magnetic fields showed increased regeneration associated with marked activation of the extracellular signal regulated kinases (ERK) and heat shock protein (hsp70). Recently we [15] found that immediately after planarian were sectioned only a single, 30 to 45 min of exposure to asymmetrically patterned, extremely low frequency magnetic fields, about the same duration shown for similar field shapes to increase activity of messenger RNA [16], produced comparable effects. Weak magnetic fields less than 1 µT accelerate oxidation of cytochrome C *in vitro*, an electron transport enzyme, and affect the functions of Na and K-ATPases [17]. None of these effects have been as reliable, conspicuous and qualitatively distinctive as the phenomenon reported here.

## Methods

### Planaria

A total of 1, 753 planarian *Dugesia tigrina* were employed as subjects; they had been obtained from different sources (North Carolina Biological Supply and Boreal Biological Supplies) and maintained according to standardized procedures [11]. During the experiments there were 15 to 30 worms per jar and 4 to 6 jars. These numbers were the same for any given block. There was the equivalent of 1 cc of spring water from Feversham, Grey County, Ontario per planarian. The length of the planarian varied between batches from 1 cm to 3 cm. However there was no difference in lengths between planarians within a given block that were exposed to the experimental and sham conditions. Ion content (ppm) was HCO_3_ 270, Ca 71, Mg 25, SO4 5.9, Cl 2.7, NO3 2.6 and Na 1. In Experiment 1 the different blocks (5) were completed in jars composed of different materials (glass, clear plastic, opaque specimen); this did not influence the effect. For all subsequent blocks the jars were glass.

### Exposure System

Two coils were used; one was a classic Helmholtz coil while the other were two rectangular coils (1.15×1.15 m). Jars were separated by 10 cm along the outer edge of the Helmholtz coil and by 40 cm (jars placed on a wooden platform) between the two rectangular coils that were separated from each other by 65 cm. The peak intensity of the FM field within the exposure area for the former was 2 µT for the FM and 5.5 µT (55 mG) for the GM field. Within the exposure area generated by the two rectangular coils the peak values for the FM field was 200 nT and 490 to 600 nT for the GM field. The equipment was the same as that employed in previous studies [17], [18].

### Magnetic Field Generation

**Figure 1:**
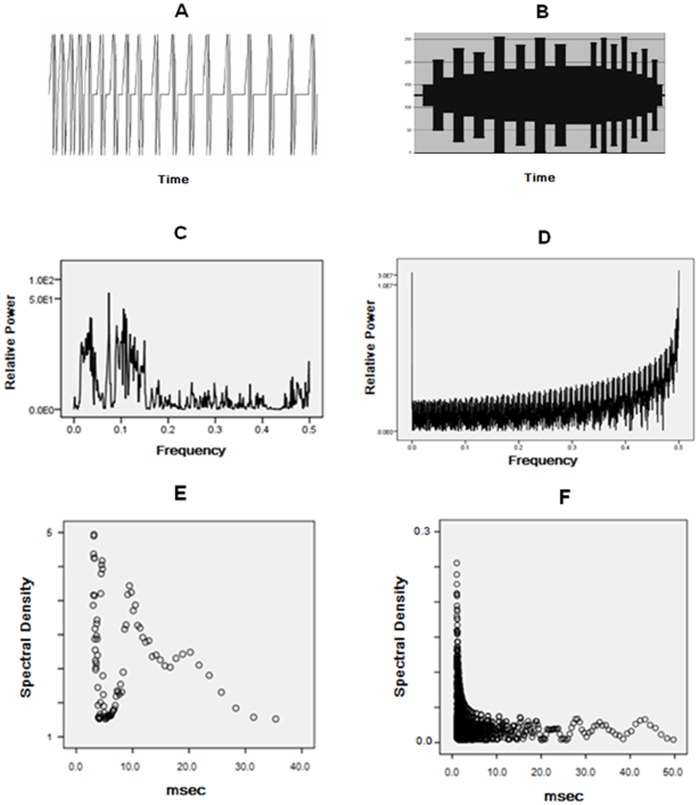
Wave form and spectral characteristics of FM and GM fields. A) the FM (“Thomas”) pulse pattern (duration 2.58 s) that was repeated continuously for 6.5 hr. for 5 consecutive days. B) an overall shape of the GM (duration = 15.3 s) pattern that was repeated continuously on the 5^th^ day for 6.5 hr. C) raw spectral analyses of FM pattern; D) raw spectral analysis of GM pattern. E) transformation of spectral power (vertical axis) to real time (accommodating the 3 ms points) of duration (inverse of frequency) of the FM pattern. F) transformation of spectral power (vertical axis) to real time for the GM pattern.

Pictorial representations of the wave pattern of the applied fields and their spectral analyses are shown in Figure 1. The FM pattern (Thomas.dac) was composed of 849 values (each between 0 and 256). It is known for its capacity to induce analgesia in several vertebrate and invertebrate species [19]–[22]. The duration each point was activated, the point duration, was 3 ms. This means that the duration of each FM sequence was 2.5 s before repeating continually for 6.5 hr and each GM sequence was 15.3 s (“65 mHz”) before repeating continually for 6.5 hr.

The GM pattern (geomagn5071.dac) was composed of 5,071 points and had been initially designed to imitate sudden geomagnetic storm commencements [23]–[25] but the point durations were reduced to 1 ms. The duration of each of first 14 wider peaks and troughs was 600 ms (200 points) while the duration of the second 14 narrow peaks and troughs was 300 ms (100 points) with an interface of 1.5 s (500 points). During this time the voltage equivalent (−5 to +5 V) of the number between 0 and 256 was converted by custom constructed digital to analogue converters (DACs) to current that was delivered to the coils. The different coils were operated by different computers each loaded with the Complex software required to produce the fields. For the wave file component of the experiment, the sound card generated a voltage from a laptop that was connected directly to the rectangular coils. The intensity within the exposure area was comparable for both the FM and GM patterns.

## Results

**Figure 2:**
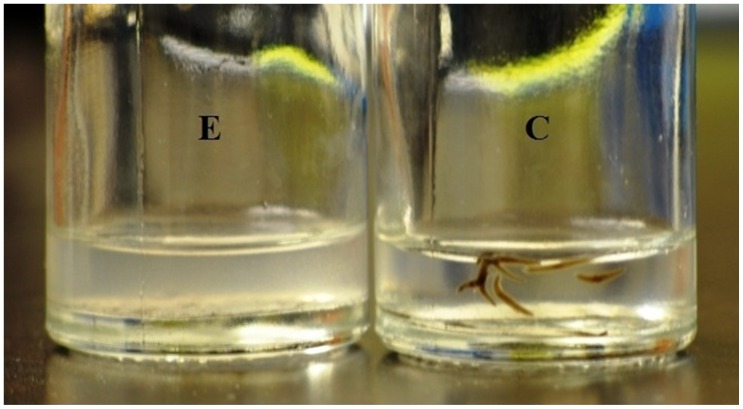
Dissolution effect on planaria exposed to FM for 5 days and successive GM exposure on 5^th^ day. Typical results following exposure to the FM and then GM weak magnetic fields after the fifth day of exposure. C is a sham field or control group of planarian within which there were never mortalities even up to two weeks later in the same environment. E refers to the dissolved debris of the same number of planarian that had been exposed to the FM-GM field combination.

The summary for all of our results are listed in Table 1. For the first series of exposures intensities averaged 2.5 µT during the FM field and 5 µT during the GM field and would be similar to those encountered near some electronic equipment. The dissolutions of the planarian were obvious within 6 hours (earliest measurement) after the initiation of the GM, conspicuous after 12 hrs (6 hrs after termination of GM) and maximum by 24 hr (12 hr after GM field termination). Over the course of their termination, planarian exposed to the FM field would expand to about (visually inspection) twice their volume. Once the GM had been applied the planaria would shrink to about half their normal size, display spasmodic contractility, become immobile and then dissolve completely with no fragments (Figure 2).

**Table 1:** Percentages of planarian (in each block of experiments) that were dissolved in the reference groups and the experimental groups as a function of time since the exposure to the final magnetic field 6 hrs and 24 hrs later.

Exposure Parameters	+6 hr		+24 hr	
	Control	Field	Control	Field
**5-d FM (2.5 µT), 1-d GM (5 µT)** **(3 ms point durations)**	0	100	0	100
	0	87	0	100
	0	80	0	100
	0	80	0	100
	0	80	0	100
**5-d FM (0.2 µT), 1-d GM(0.5 µT)**	0	35	0	100
	0	46	0	100
	0	39	0	100
**5-d GM, 1-d GM (GM only)**	0	0	0	0
	0	0	0	0
	0	0	0	0
**5-d FM, 1-d FM (FM only)**	0	0	0	0
	0	0	0	0
	0	0	0	0
**5-d GM, 1-d FM (reverse)**	0	0	0	0
	0	0	0	0
	0	0	0	0
**1-d FM, 1-d GM**	0	0	0	0
	0	0	0	0
**3-d FM, 1-d GM**	0	64	0	100
	0	68	0	100
**5-d FM, 1-d GM** **(5 ms point durations)**	0	0	0	0
	0	0	0	0
	0	0	0	0
**5-d FM, 1-d GM (wave files)**	0	0	0	0
	0	0	0	0
	0	0	0	0

The effective parameters were the FM field for 6.5 hr per day for 5 days and on the fifth day 6.5 hr exposure to the GM field as well.

The effect was *not* apparent if the jars containing the planarian were moved from or disturbed in exposure area or ambient (600 lux) light was present. The phenomena were apparent when the experiments were performed in the dark or in ambient light less than ∼10 lux. This “dark” dependence was not considered unusual considering the light-attenuating effects of extremely low frequency magnetic fields upon attenuation of opioid analgesia in mice [26] and nitric oxide activation in the land snail [27].

To discern the reliability of this robust phenomenon we exposed jars of planarian (10 to 15 planarian per jar) either to the magnetic field configuration (FM-GM) generated within a traditional Helmholtz coil or sham-field conditions. For 5 blocks (one block per week) all 332 planarian that had been exposed to the configuration were dissolved within 24 hr while none of the 236 planarian in the control conditions died. Within six hours after the onset of the GM field the percentage of worms that had dissolved in the 5 blocks, as discerned by visual inspection (without moving the jars) were 100%, 87%, 80%, 80% and 80% respectively. The results of this component of the experiment as well as the blocks of all the experiments are presented in Table 1 to facilitate clarity.

To insure that the effect could be produced by other equipment, the procedure was repeated with a different computer and a much larger coil system [11] where the intensity of the FM field was 0.2 µT and the GM field was about 0.5 µT. These three blocks of experiments were completed in the basement (completely dark) in another building. Again, within 24 hr after the initiation of the GM field after 5 days of exposure to the FM field 100% of the planarian exposed to the configuration was dissolved. For comparison with the first experiment, after 6 hrs between 35% and 46% of the configuration field-exposed planarian had dissolved while none of the planarian in the control group dissolved.

In all subsequent blocks of experiments there were 15 worms per jar and 3 jars in the field condition and one jar in the control condition per block. We then altered the presentation of different components of the configuration. In three separate blocks, *only* the GM field was presented (instead of the FM field) for 6.5 hr per day for 5 days and then for an extra 6.5 hr on the 5^th^ day. Three other blocks were conducted where only the FM field was presented for 6.5 hr per day and then for an additional 6.5 hr on the 5^th^ day in order to discern if the terminal GM component was essential. There were no mortalities for either condition. The reversed presentation of the effective sequence, that is the GM field for 5 days for 6.5 hr per day followed by the addition of 6.5 hr on the fifth day, also produced no mortality. These results strongly suggested that the precise sequence of the FM field first followed by the GM field was the necessary condition to produce the 100% mortality of planarian within 24 hr. None of the control jars containing 230 planarian, involving nine blocks, died and were still viable 10 days after the end of the experiment before they were discarded.

To establish the threshold for the temporal duration required to produce the mortality, planarian were exposed for one day (6.5 hr) to the FM field and then immediately to the GM field for 6.5 hr; there was no mortality. Following three days of daily exposure to the FM field and then to the GM field (two blocks) the average mortality was 66%. This suggested that more than one daily exposure and at least 3 days of exposure were required to start the effect and that 5 days was sufficient to produce 100% mortality.

Both the FM and GM patterns of magnetic fields were generated by computer software that converted columns of numbers (849 for the FM and 5,071 for the GM) to appropriate voltages to generate the magnetic fields in the same space within which the planarian were placed. The point duration of each number between 0 and 256 was programmable in ms. We had selected 3 ms as the point duration (the time each voltage is presented through the circuit to the coil) because of its demonstrated efficacy for analgesia [4] for the FM field when presented to rodents and for its retarding effect upon the growth of several types of cancer cells but not normal (mouse and human) cells for one-hour daily presentations for five days [29]. In the latter *in vitro* setting the same FM pattern employed in this study but presented for one hour per day was not effective when the point durations were either 1, 2, 4, or 5 msec.

To test this application in the present context, planarians were exposed to the same configuration but the point duration was changed from 3 ms to 5 ms for 3 blocks. There were no mortalities. We then copied the magnetic pattern generated from the large coil to a wave file. The fidelity of the pattern was established by listening to the auditory output produced from the magnetic field by a magnetic sensor (solenoid) coupled to an acoustic amplifier. We employ several of these devices in the laboratory routinely to insure the presence and temporal structure of magnetic fields employed in a variety of studies. Planarian exposed to the wave file version of the configuration rather than the one generated by the digital-to-analogue transformation from the complex software exhibited no mortality.

We decided to visualize the phenomena over time by recording the exposed planarian’s movements during the 24 hr following the onset of the GM field. An infrared camera recording 1,000 frames per sec of 3 jars (each containing 15 planarians) recorded activity of the planarian for 6.5 hr after the onset of the GM. Within 15 min following the activation of the GM field, the planarian moved more frequently. After 60 min the planarian displayed twisting and contortion movements and no longer adhered to the side of the jars. After 6 hr the planarian no longer ascended to the surface. Within 24 hr all of the planarian were dead and dissolved. A time-lapsed video of this progression is available.

## Discussion

We have completed several studies involving planarian in various types and intensities of magnetic fields [29]. The present observation of death and complete dissolution of planarian exposed to this combination of fields is unprecedented in our observations as well as the general literature. Qualitatively, it appears that the exposure to the FM field may weaken the structural protein that maintains the organism resulting in a visually obvious increase in body volume of the planarian followed by rapid contraction and dissolution of the boundary between the ambient water and the intraorganismic fluid after GM field onset. During a subsequent series of unpublished experiments involving mouse B16 melanoma cells, the same exposure paradigm employed in the present study that produced dissolution of the flatworms resulted in fragmentation of the melanoma cells [28]. Within 5 hr of the exposure to the GM field there were no discernable intact cells with the cultures that had been exposed to the procedure. Visually obvious enlargement followed by shrinkage of these cells within a similar time frame was also observed.

Although the role of calcium influx [30], [31] membrane resonance [32], opening of calcium and potassium channels [11] coupled to melatonin receptors [33] (the light-sensitive ligand for which is known to be responsive to multiple forms of weak time-varying magnetic fields [34] and direct effects on DNA [35], [36] are the most parsimonious sources of mechanism, a yet to be identified process involving coherent domains of water [37], [38] during exposure to the fields may be critical. If the temporal pattern is critical to the phenomenon then the “dissolution effect” might occur during exposures to field intensities even less (<100 nT) than the ones employed in this study. It is relevant that the magnetic fields that produced this effect are not sine-wave or symmetrical patterns and were generated by computer software rather than function generators.

## Supporting Information

Video S1 **A time-lapsed film of the activity of the planarian after initiation of the GM field can be found at the specified address.**
(WMV)
